# Assessment of NSAIDs as potential inhibitors of the fatty acid amide hydrolase I (FAAH-1) using three different primary fatty acid amide substrates in vitro

**DOI:** 10.1186/s40360-021-00539-1

**Published:** 2022-01-04

**Authors:** Julius T. Dongdem, Gideon K. Helegbe, Kwame Opare-Asamoah, Cletus A. Wezena, Augustine Ocloo

**Affiliations:** 1 Department of Biochemistry and Molecular Medicine, School of Medicine, University for Development Studies, Tamale-Campus, Tamale, Ghana; 2 School of Life Sciences, University of Nottingham Medical School, NG7 2UH Nottinghamshire, UK; 3 Department of Physiology and Biophysics, School of Medicine, University for Development Studies, Tamale-Campus, Tamale, Ghana; 4 Department of Microbiology, Faculty of Bioscience, University for Development Studies, Nyankpala Campus, Tamale, Ghana; 5 Department of Biochemistry, Cell and Molecular Biology University of Ghana, Legon, Accra, Ghana

**Keywords:** Arachidonamide, Affinity, FAAH-1, Hydrolysis, Oleamide, Arachidonamide, Stearoylamide, Inhibition, NSAIDs, Mode

## Abstract

**Background:**

Pain relief remains a major subject of inadequately met need of patients. Therapeutic agents designed to treat pain and inflammation so far have low to moderate efficiencies with significant untoward side effects. FAAH-1 has been proposed as a promising target for the discovery of drugs to treat pain and inflammation without significant adverse effects. FAAH-1 is the primary enzyme accountable for the degradation of AEA and related fatty acid amides. Studies have revealed that the simultaneous inhibition of COX and FAAH-1 activities produce greater pharmacological efficiency with significantly lowered toxicity and ulcerogenic activity. Recently, the metabolism of endocannabinoids by COX-2 was suggested to be differentially regulated by NSAIDs.

**Methods:**

We analysed the affinity of oleamide, arachidonamide and stearoylamide at the FAAH-1 in vitro and investigated the potency of selected NSAIDs on the hydrolysis of endocannabinoid-like molecules (oleamide, arachidonamide and stearoylamide) by FAAH-1 from rat liver. NSAIDs were initially screened at 500 μM after which those that exhibited greater potency were further analysed over a range of inhibitor concentrations.

**Results:**

The substrate affinity of FAAH-1 obtained, increased in a rank order of oleamide < arachidonamide < stearoylamide with resultant V_max_ values in a rank order of arachidonamide > oleamide > stearoylamide. The selected NSAIDs caused a concentration-dependent inhibition of FAAH-1 activity with sulindac, carprofen and meclofenamate exhibiting the greatest potency. Michaelis-Menten analysis suggested the mode of inhibition of FAAH-1 hydrolysis of both oleamide and arachidonamide by meclofenamate and indomethacin to be non-competitive in nature.

**Conclusion:**

Our data therefore suggest potential for study of these compounds as combined FAAH-1-COX inhibitors.

**Supplementary Information:**

The online version contains supplementary material available at 10.1186/s40360-021-00539-1.

## Introduction

Several therapeutic agents have been designed to address different forms of pain, yet pain relief remains an area of significant unmet patient need [1, 2]. Drugs administered to treat pain and inflammation presently have low to moderate efficiencies with significant untoward side effects such as gastrointestinal bleeding, ulceration, renal dysfunction, nausea and vomiting.

Fatty acid amide hydrolase I (FAAH-1) has been proposed as a promising target for the discovery of drugs to treat pain, inflammation and other pathologies [3, 4]. FAAH-1 is the primary enzyme that is responsible for the degradation of N –Arachidonoyl ethanolamide (Anandamide, AEA) and related fatty acid amides which constitute a group of biologically active endogenous amides [5, 6]. Inhibition of FAAH-1 results in the accumulation of AEA and other endocannabinoid-like molecules in the central and peripheral nervous systems where they act as ligands of cannabinoid (CB_1_ and CB_2_) receptors. Similar to Δ9 -tetrahydrocannabinol (THC), AEA is a partial agonist at both CB_1_ and CB_2_ transmembrane receptors - members of the G-protein-coupled receptor superfamily [7–9] however, in contrast to THC, AEA also stimulates the transient receptor potential vanilloid receptor type 1 (TRPV1) [10–12]. AEA exhibits cannabimimetic effects at the cannabinoid receptors [13]. Palmitoyl ethanolamide has also been reported to be active at peroxisome proliferator-activated receptors (PPARs) as well as vanilloid receptors. The primary fatty acid amides (PFAMs) such as oleamide, arachidonamide, stearoylamide, stearoyl ethanolamide, palmitamide, etc.) are also important molecules controlling sleep, angiogenesis, locomotion, convulsions and inhibition of gap junction formation among several other functions [14–18].

Although the major current strategy for drug development is to design compounds that are selective for a given target, compounds that target more than one biochemical process may have superior efficacies with better safety profiles compared with standard selective compounds. This can be achieved by administering the drugs either separately or in single tablets made of more than one active ingredient. The disadvantage in both cases is the potential for a large pharmacokinetic variability that is equivalent to the concomitant administration of separate drugs. The alternative to avoid these drawbacks is to develop drugs that target more than one molecular mechanism [19]. Inhibition of COX-1 and -2 at the first committed step of prostanoid and other eicosanoid biosynthesis from arachidonic acid (AA) underlies the analgesic action of non-steroidal anti-inflammatory drugs (NSAIDs) [20–23]. NSAIDs constitute a class of chemically diverse compounds that provide analgesic, antipyretic and anti-inflammatory effects. The fatty acid metabolic end-products of the induction of the COX cascade by a wide range of stimuli are prostaglandins (PGD_2_, PGE_2_, PGF_2α_ and PGI_2_). AA embedded in cell membranes as esters of phospholipids is the precursor of prostaglandins (PGs). AA is made available by action of several enzymes including cPLA_2_/sPLA_2_, αβ Hydrolase 4 and GDE [24]. Once induced, COX, LOX and cytochrome P450 enzymes convert available AA to various eicosanoids. These eicosanoids are known essential physiological and pathophysiological mediators implicated in a wide scope of therapeutic interest such as in inflammation, pain, cancer, glaucoma, male sexual dysfunction, osteoporosis, cardiovascular disease, labour, asthma, etc [25]

Selected NSAIDs have also been reported to inhibit FAAH-1 activity from mouse and rat preparations [26]. Studies in animal models have revealed that the simultaneous inhibition of COX and FAAH-1 activities produce greater pharmacological efficiency with significantly lowered toxicity and ulcerogenic activity associated with COX inhibitors [27, 28]. More recently, the metabolism of endocannabinoids by COX-2 was suggested to be differentially regulated by NSAIDs resulting in antinociceptive effects mediated via cannabinoid receptors [29–32]. Apart from catalysing the formation of PGs from AA, COX-2 also catalyses the formation of prostaglandin-glycerol esters and prostaglandin ethanolamines from 2-arachidonoyl glycerol (2-AG) and AEA respectively [30, 33–35]. Since COX-2 is a significant target of NSAIDs, COX-2 inhibition can reduce this mechanism of endocannabinoid metabolism to enhance their concentrations in vivo [36, 37]. Moreover, rapid reversible inhibitors of COX-2 selectively inhibit the oxygenation of 2-AG and AEA with much higher potencies for AA, a phenomenon referred to as substrate selective effect [30, 38]. The fact that selected NSAIDs inhibit AEA and 2-AG metabolism via FAAH-1 and COX inhibition in vivo, suggests that at the appropriate concentrations, NSAIDs may co-regulate the activity of both COX and FAAH-1 enzymes which make them better suitable therapeutic agents [39, 40]. Since cannabinoids possess anti-inflammatory, antinociceptive, analgesic, anti-tumour and immunosuppressive properties, inhibitors of endocannabinoid degrading enzymes (FAAH-1, FAAH-2, NAAA, COX-2, LOX, MAGL) may be of therapeutic significance via augmentation of endocannabinoid and endocannabinoid-like molecule accumulation in vivo. Based on this previous knowledge, it is essential to conduct further investigations on the ability of other NSAIDs to inhibit FAAH-1 deamination of endocannabinoid and endocannabinoid-like molecule substrates (e.g. oleamide, arachidonamide, stearoylamide and stearoyl ethanolamide among others) for the reason that NSAIDs with both inhibitory capabilities (on COX and FAAHs) will synergistically enhance therapeutic efficacies. The aim of this study therefore, was to assess pharmacological profiles of FAAH-1 with regards to potential substrates and inhibitors. The investigation was specifically designed to assess the potency of selected NSAIDs on the hydrolysis of oleamide, arachidonamide and stearoylamide by FAAH-1.

## Materials and methods

FAAH-1 activity was studied in rat liver homogenate.

### Preparation of rat liver homogenate

Liver obtained from male Wister rats (150–250 g, Charles River Laboratories, Wilmington, USA) which had been stored at − 40 °C was thawed. A volume of 6 ml/g wet weight of rat liver was homogenized in 0.2 M potassium phosphate buffer, pH 7.4 using a hand held homogenizer (Ultra-turrax) (Merck KGaA, Darmstadt, Germany). The resulting mixture was centrifuged at 250 g for 10 min after which the pellet obtained was re-homogenised and centrifuged as aforementioned. The supernatants were combined and centrifuged at 20,000 g for 30 min, after which the membrane containing pellet was re-suspended in 1:1^w^_/v_ 0.2 M potassium phosphate buffer, pH 7.4, and stored in 1 ml aliquots at − 40 °C.

### Assay of FAAH-1 activity

FAAH-1 activity was assayed essentially as described previously [41]. Briefly, rat liver homogenate was pre-incubated at 37 °C with shaking (50 × 10 rpm) for 10 min in 0.2 M phosphate buffer, pH 7.4 in 96-well microtitre plates (Thermo Scientific Inc., Waltham, USA) prior to substrate addition and incubation at 37 °C for 30 min. The 100 μl total assay reaction mixtures were halted with an equivalent volume of o-phthaldehyde (OPA) developing solution (0.4 M potassium phosphate buffer, pH 11.5) and incubated further at 37 °C for 15 min before assessing fluorescence using a FLUOstar Galaxy (Excitation 390 nm, Emission 450–10 nm) (BMG LABTECH GmbH, Ortenberg, Germany). Substrate blank and a control containing 0.2 M phosphate buffer, pH 7.4, were incorporated into the experiments.

Subsequently, the influence of ethanol concentrations on the ability of particular NSAIDs e.g. indomethacin (SIGMA-ALDRICH, Poole, UK) was assessed by varying the volume of inhibitor solution added, using both absolute ethanol and buffer blanks to account for background influences on enzymatic activity.

### Protein assay

Homogenate protein content was measured by modifications of the method described [42] using 200 μl of different concentrations of bovine serum albumin (0, 25, 50, 100, 150, 200, 300 μg/ml) as standard and 200 μl of 0.5 M NaOH as blank (Fig. SS1). Briefly, 50 μl of each membrane fragment in 5 ml of 0.5 M NaOH was prepared, after which 200 μl of each dilution was added to 1 ml of solution A (100 ml of 2% sodium carbonate and 1 ml each of both sodium potassium tartrate and copper sulphate). The solutions were mixed and allowed to stand at room temperature. After 10 min, 100 μl of dilute Folin Ciocalteau’s reagent 1:1 ddH_2_O was added and mixed immediately. The absorbance of each sample was read at a wavelength of 700 nm following incubation at room temperature for 1 h. Relative absorbance of each sample was entered into GraphPad prism and analysed. The protein concentration of preparations were interpolated from the standard (Fig. SS1), using non-linear, second order polynomial (quadratic) graph of the standards.

### Statistical analysis

Data obtained were entered into a Microsoft Excel 2010 spread sheet and analysed with GraphPad Prism computer software programme (GraphPad Software Inc., San Diego, CA USA). Effect of 500 μM concentration of NSAIDs (SIGMA-ALDRICH, Poole, UK) on each enzyme activity was analysed by removing the baseline line. Each specific activity was then plotted as percentage of control. Specific activity obtained at each inhibitor concentration for the concentration-inhibition curves were normalized and analysed using the inbuilt log (inhibitor) versus response variable slope (robust fit) and were constrained at the bottom (= 0.0%). Each specific activity was then plotted as percentage of the control. To determine the mode of inhibition, V_max_ values were initially extrapolated from the (NH_4_)_2_SO_4_ standard curve plotted using the inbuilt second order polynomial (quadratic) Michaelis-Menten enzyme kinetics. These values were then adjusted using the protein concentrations of the preparations obtained from the Lowry protocol [42] (Fig. SS1 and SS2).

## Results and discussion

### Affinity of oleamide, arachidonamide and stearoylamide at FAAH-1

**Fig. 1:**
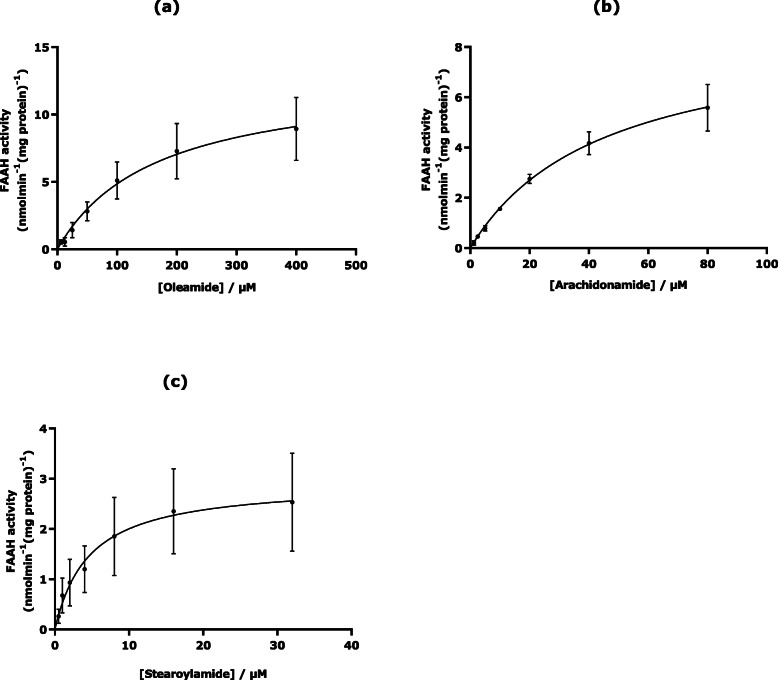
Hydrolysis of oleamide (**a**), arachidonamide (**b**) and stearoylamide (**c**) by rat liver FAAH-1 activity. Rat liver FAAH-hydrolytic activity of each primary amide substrate in vitro, was assayed by quantification of ammonia released after hydrolysis. Ammonia generated in the presence of sulphite ions is reacted with alkaline o-phthaldehyde (OPA) to generate the stable fluorescent isoindole derivative (1-sulphonatoisoindole) which is quantified by fluorescent spectroscopy [41, 43]. Four separate experiments with three replicates on the same microtiter plate were conducted for each substrate using different rat liver preparations. Data are mean ± SEM (Standard Error of the Mean) of four separate preparations (*n* = 4) conducted in triplicate

**Fig. 2:**
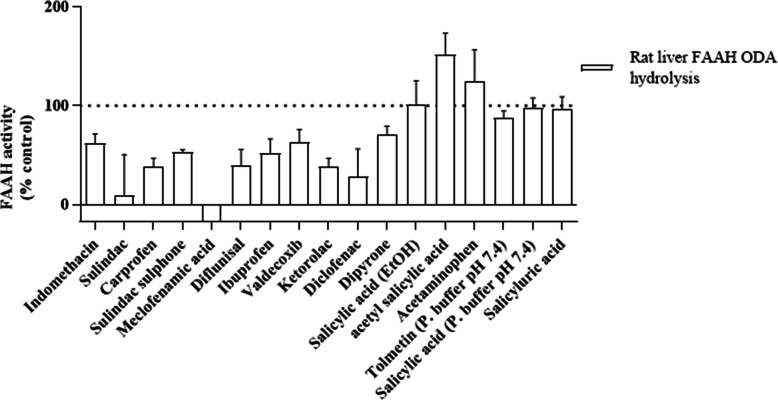
Effect of 500 μM concentration of NSAIDs on rat liver FAAH-1 oleamide hydrolase activity. Data are mean ± SEM (Standard Error of the Mean) of four separate preparations (*n* = 4) conducted in triplicate

Several drugs are inhibitors of the most relevant enzymes since blocking these enzymes can kill a pathogen or correct a metabolic imbalance. To characterise an enzyme in the presence of inhibitors however, a good kinetic description of its activity is essential. Here, the ability of rat liver to hydrolyse oleamide, stearoylamide and arachidonamide was assessed by Michaelis-Menten analysis (Fig. 2). The resultant Michaelis-Menten constant (K_m_) and maximum velocity (V_max_) values obtained are summarized in Table 1. The substrate affinity of FAAH-1 increased in a rank order of oleamide < arachidonamide < stearoylamide with resultant V_max_ values in a rank order of arachidonamide > oleamide > stearoylamide (Fig. 1, Table 1). The kinetic values for FAAH-1 hydrolysis of oleamide obtained are consistent with previous observations. Similar K_m_ and V_max_ values of 129 μM and 15 nmol.min^− 1^.mg protein^− 1^ from oleamide hydrolysis by FAAH-1 in rat liver preparations and a K_m_ value of 179 μM with FAAH-1 in rat brain were previously obtained compared with K_m_ of 177.2 ± 15.5 μM and V_max_ of 8.9 ± 1.1 nmol.min^− 1^.mg protein^− 1^ obtained in our findings (Table 1) [41]. An affinity of 104 μM and a V_max_ of 5.7 nmol.min^− 1^.mg protein^− 1^ for rat liver FAAH-catalysed oleamide hydrolysis has been reported [44]. Additionally, an affinity of 37 ± 7 μM at pH 9 for rat recombinant FAAH-catalysed oleamide hydrolysis has also been reported [45].

**Table 1:** K_m_ and V_max_ values determined for rat liver FAAH-1 hydrolysis of three different fatty acid amides

FAAH-1 kinetics	Substrate		
	Oleamide	Arachidonamide	Stearoylamide
**K**_**m**_ **(μM)**	177.2 ± 15.5	44.9 ± 7.0	4.6 ± 0.8
**V**_**max**_ **(nmol/min/mg protein)**	8.9 ± 1.1	10.1 ± 3.0	2.5 ± 0.6

Data are mean ± SEM (Standard Error of the Mean) of four separate preparations (*n* = 4) conducted in triplicate

FAAH-1 has the ability to hydrolyse a wide range of unsaturated and, to a lesser extent, saturated PFAMs and other fatty acids e.g. oleamide and palmitoyl ethanolamide [46, 47]. In our findings, FAAH-1 capacity (V_max_) was 12% higher for arachidonamide compared with oleamide and 75% higher than that for stearoylamide. This confirms the propensity of FAAH-1 to turn over polyunsaturated PFAMs particularly with *cis* double bonds at higher rates than monounsaturated and saturated PFAMs and is consistent with literature (Fig. 1) [48, 49].

### Screening of NSAIDs as potential inhibitors of oleamide, arachidonamide and stearoylamide hydrolase activity

Following pilot experiments that revealed indomethacin to have an IC_50_ ~ 500 μM, 16 selected NSAIDs were screened at 500 μM (Fig. 2) for ability to inhibit FAAH-1 in order to assess pharmacological profiles of rat liver FAAH-catalysed hydrolysis of the three PFAMs assayed at a concentration ≥ K_m_ value determined [41, 43]. NSAIDs were randomly selected based on availability and considering what had not been reported while using a few that had been reported against FAAH-1 as reference standards. Meclofenamic acid exhibited complete inhibition of FAAH-1 activity when oleamide was used as substrate. Sulindac, diclofenac, carprofen, ketorolac and diflunisal exhibited a higher degree of inhibition of rat liver FAAH-1 activity by inhibiting oleamide hydrolysis to below 50% of control (Fig. 2). Ibuprofen, sulindac sulphone, indomethacin and dipyrone were moderate inhibitors of oleamide hydrolysis and inhibited FAAH-1 activity to between 50 and 70% of control. Tolmetin, salicyluric acid, salicylic acid (diluted in 0.2 M potassium phosphate buffer) evoked weak inhibitory ability of FAAH-1 activity to between 70 and 100% of control. Acetaminophen and acetyl salicylic acid appeared to enhance enzyme activity.

Acetaminophen is reported to be metabolised to N-arachidonoylaminophenol (AM404) via FAAH-1 [50]. AM404 then inhibits FAAH-1 activity and prevents AEA metabolism. Thus, FAAH-1 is active until concentrations of AM404 are high enough to inhibit its function. AEA accordingly activates platelets, however, the process is unaffected by acetyl salicylic acid, thus it is possible it did not affect rat liver FAAH-1 activity [51]. The differences in reaction of FAAH-1 to specific compounds (e.g. ketorolac or ibuprofen) might be due to differences in structures, their sites of binding to FAAH-1 and how this affects substrate entry and binding at the catalytic sites [52–55].

### Effect of vehicle controls on FAAH activity

**Fig. 3:**
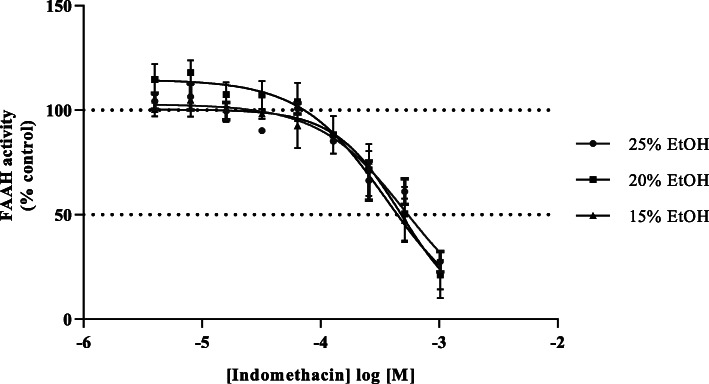
Effect of 15, 20 and 25% ethanol on the inhibition of rat liver oleamide hydrolase activity by indomethacin. Data are mean ± SEM (Standard Error of the Mean) of four separate preparations (*n* = 4) conducted in triplicate

As the NSAIDs are differently soluble in aqueous compared to organic solution, the effect of a range of concentrations of the vehicle ethanol was assessed using indomethacin as a reference compound. Indomethacin evoked a concentration-dependent inhibition of FAAH-1 activity in pIC_50_ values between 15, 20 or 25% ethanol concentrations (Fig. 3). Tukey’s multiple comparisons test with single pooled variance, *p* = 0.7250, *p* < 0.05 as significantly different, CI = 95% indicated no significant difference between pIC50 values obtained (Table 2). This implies that, within the experimental limits, ethanol had no effect on the inhibitory function of indomethacin, albeit with a reduced capacity for basal oleamide hydrolysis of 95 ± 1, 78 ± 1 and 76 ± 4% of control for 15, 20 and 25% assay ethanol respectively consistent with earlier reports that butanol reduced FAAH-1 activity by 30 to 50% but did not affect the enzyme response to inhibitors [40].

**Table 2:** Potency of indomethacin in the presence of different concentrations of ethanol

	15% EtOH	20% EtOH	25% EtOH
pIC50	3.4 ± 0.1	3.5 ± 0.1	3.4 ± 0.1
FAAH-1 activity (%)	95 ± 1	78 ± 1	76 ± 4%

Data are mean ± SEM (Standard Error of the Mean) of four separate preparations (*n* = 4) conducted in triplicate

### Concentration-dependence of rat liver FAAH-1 oleamide hydrolase inhibition

**Fig. 4:**
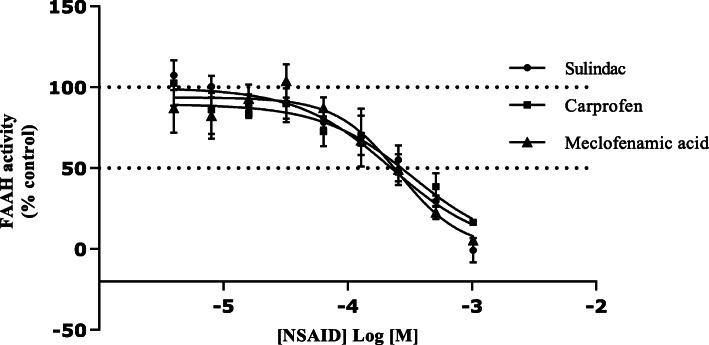
Concentration-dependence of rat liver oleamide hydrolase activity inhibition. Data are mean ± SEM (Standard Error of the Mean) of four separate preparations (*n* = 4) conducted in triplicate

NSAIDs selected on the basis of the greater levels of inhibition at 500 μM were examined over a range of concentrations in absolute ethanol, from 4.0 × 10^− 6^ to 1.024 × 10^− 3^ M (Fig. 4). These exhibited concentration-dependent inhibition of FAAH-1 oleamide hydrolase activities. The order of inhibitory potency against rat liver FAAH-1 hydrolysis of oleamide was sulindac > carprofen > meclofenamic acid > sulindac sulphone > indomethacin > diflunisal > ibuprofen > valdecoxib > ketorolac > diclofenac > dipyrone (Table 3). The remaining NSAIDs assayed exhibited very similar potencies (pIC_50_ values) against activity of FAAH-1. The inhibition exhibited by the selected NSAIDs to FAAH-1 activity (Fig. 4, Table 3) is consistent with earlier studies although under different conditions [26, 39, 56, 57]. The rank order of potency displayed by NSAIDs screened at 500 μM was not exactly the same when the pIC_50_ values were examined. Earlier findings indicate that NSAID inhibition of FAAH-1 activity is pH dependent [58] with a pH optimum of ~ 9 [46, 59–63]. The rank order of NSAIDs reported for potency against rat brain FAAH-1 activity at pH 7.4 was; indomethacin (pIC_50_ = 4.18) ≈ carprofen (pIC_50_ = 4.10) > ibuprofen (pIC_50_ = 3.1) and is similar to our findings however, indomethacin was less effective than carprofen and more potent than ibuprofen [52]. Other studies found apparently biphasic pH dependence of FAAH AEA metabolism using brain microsomes [64].

**Table 3:** Potencies of NSAIDs as inhibitors of rat liver oleamide hydrolase activity

NSAID	pIC50 (M)	NSAID	pIC50 (M)
Sulindac	3.65 ± 0.08	Ibuprofen	3.01 ± 0.06
Carprofen	3.58 ± 0.09	Valdecoxib	3.00 ± 0.15
Meclofenamic acid	3.57 ± 0.06	Ketorolac	2.91 ± 0.07
Sulindac sulphone	3.35 ± 0.03	Diclofenac	2.90 ± 0.07
Indomethacin	3.28 ± 0.03	Dipyrone	2.77 ± 0.07
Diflunisal	3.15 ± 0.04		

Data are mean ± SEM (Standard Error of the Mean) of four separate preparations (*n*=4) conducted in triplicate

### Mode of inhibition of FAAH-1 metabolism by meclofenamic acid and indomethacin

To date, little has been reported on the mode of inhibition of NSAIDs on FAAH-catalysed hydrolysis of endocannabinoids and endocannabinoid-like molecules [26, 52]. Hence, meclofenamic acid and indomethacin were selected for further mechanistic investigation as the former evoked the greatest inhibition and the latter has previously been examined extensively in the literature [26].

**Fig. 5:**
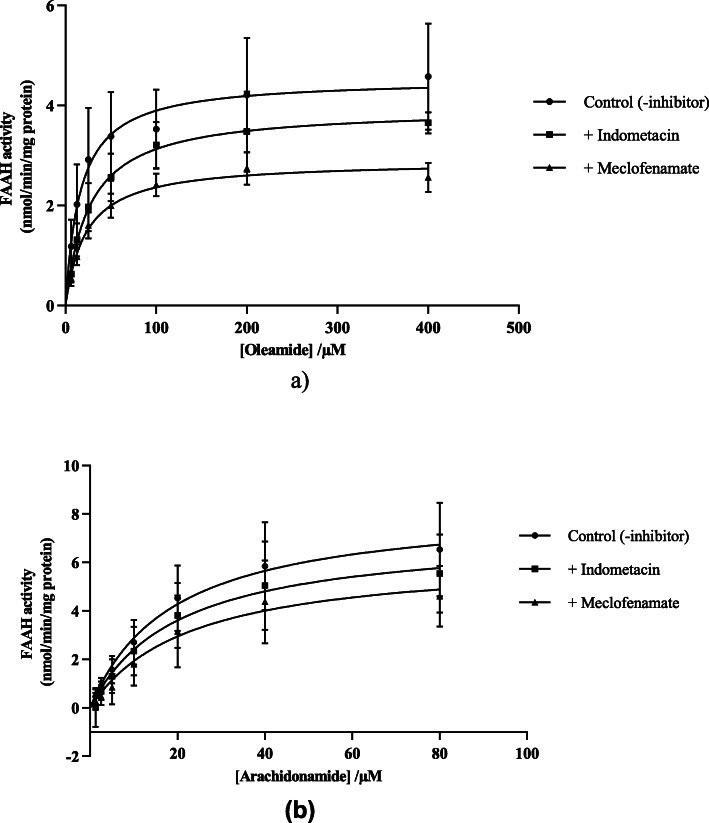
Mode of inhibition of rat liver FAAH-1 hydrolysis of (**a**) oleamide and (**b**) arachidonamide by meclofenamic acid and indomethacin. Data are mean ± SEM (Standard Error of the Mean) of four separate preparations (*n* = 4) conducted in triplicate

Michaelis-Menten analysis indicated no significant changes in substrate affinity (K_m_) values but with decreasing V_max_ values (Fig. 5, Table 4), thus indicative of non-competitive type inhibition of FAAH activity by the two inhibitors (meclofenamic acid and indomethacin). This finding is consistent with similar findings that FAAH is mechanistically allosteric in nature which is often associated with a non-competitive mode of inhibition, thus FAAH might also likely exhibit a non-competitive mode of inhibition against these NSAIDs [58, 65, 66]. Unlike aspirin which is an irreversible inhibitor of COX enzymes, most other NSAIDs are reversible competitive inhibitors of the COX enzymes [67]. Previously scientists [38] found that meclofenamic acid and ibuprofen are also potent inhibitors of COX-2 suggestive of the potential for the design of a dual targeting inhibitor possibly in combination with URB597 an uncompetitive FAAH inhibitor [68], which may reduce the loading dose of NSAIDs with resultant fewer side effects.

**Table 4:** Mode of inhibition of rat liver FAAH-1 oleamide hydrolysis by indomethacin and meclofenamate

FAAH-1 Kinetics	K_**m**_ (μM)	V_max_ (nmol/min/mg protein)	Substrate
Control	18.4 ± 3.5	4.6 ± 0.5	Oleamide
+ 200 μM indomethacin	24.4 ± 3.3	3.8 ± 0.4	Oleamide
+ 100 μM meclofenamate	22.7 ± 1.4	2.9 ± 0.1	Oleamide
Control	19.8 ± 2.0	8.4 ± 1.2	Arachidonamide
+ 200 μM indomethacin	21.6 ± 2.8	7.2 ± 0.9	Arachidonamide
+ 100 μM meclofenamate	23.4 ± 3.7	6.7 ± 1.0	Arachidonamide

Data are mean ± SEM of triplicate assessments conducted on five transient transfects (*n* = 5)

### Therapeutic application of novel multi-target (FAAH/COX) analgesics

In vivo increases in the levels of AEA resulting from FAAH-1 inhibition potentiates actions of COX inhibitors [19, 31] suggesting that, compounds that inhibit both FAAH and COX enzymes can be as effective as NSAIDs but with a reduced COX inhibitor ‘load’, consequently with accompanying reduction in the adverse effects associated with NSAIDs [19]. There is evidence to support the controversy that dual-action FAAH-COX inhibitors may be more useful in this aspect. In vitro evidence suggests that the metabolism of AEA by COX-2 might be the most predominant degradation pathway after blocking the major FAAH metabolic pathway. Combinations of URB597 and diclofenac have demonstrated synergistic analgesic interactions [27, 69]. Also, in vivo synergistic effect was achieved by administration of a combination of AEA and rofecoxib. Local injection of AEA with NSAID (ibuprofen or rofecoxib) generated higher amounts of fatty acid ethanolamides [70]. Synergistic effects have also been reported after a systematic administration of URB597 and diclofenac in a mouse model of visceral pain [71]. Meclofenamic acid, carprofen and indomethacin are among the most potent inhibitors of the COX enzymes and at the same time FAAH-1 from our study [72–75]. Our in vitro results support the possibility of combined therapeutic agents being explored. This suggests that, a combination of FAAH inhibitors such as URB597 and the NSAIDs with dual inhibitory capability may have greater utility to treat pain with reduced NSAID load and may have enhanced efficacies and safety profiles.

## Conclusion

We established inhibitory potencies of NSAIDs against rat liver FAAH-1 using oleamide, arachidonamide and stearoylamide as substrates. Substrate affinity of FAAH-1 increased in a rank order of oleamide < arachidonamide < stearoylamide with resultant V_max_ values in a rank order of arachidonamide > oleamide > stearoylamide. Our Findings confirmed the propensity of FAAH-1 to turn over polyunsaturated PFAMs particularly with *cis* double bonds at higher rates than monounsaturated and saturated PFAMs. In the presence of meclofenamate or indomethacin, Michaelis-Menten analysis suggested a reduction in the V_max_ of oleamide and arachidonamide hydrolysis, without significant alteration in substrate affinity, indicative of a non-competitive action of these inhibitors against FAAH-1 activity though more research is required for conclusive evidence. Even though, there was no indication of any selective action of NSAIDs, these results suggest potential for study of these compounds as combined FAAH-COX inhibitors.

## Supplementary Information

**Additional file 1.**

## Data Availability

The datasets used and/or analysed during the current study are available from the corresponding author on reasonable request.
