# Supplementary material for: Assessment of NSAIDs as potential inhibitors of the fatty acid amide hydrolase I (FAAH-1) using three different primary fatty acid amide substrates in vitro
Source: BMC Pharmacol Toxicol. 2022 Jan 4;23:1. doi: 10.1186/s40360-021-00539-1 (PMC8725537; doi:10.1186/s40360-021-00539-1)
Supplement: Supplementary file 1 — Additional file 1. [file 40360_2021_539_MOESM1_ESM.docx]

**Supplementary sheet:**

**Fig SS1: Representative standard Lowry curve for determination of protein content.**

**Fig SS2: Representative (NH_4_)_2_SO_4_ standard curve for determination of amount of ammonia generated by FAAH hydrolysis of substrates.**
